# First Trimester *Listeria monocytogenes* Septicemia

**DOI:** 10.1155/S1064744997000367

**Published:** 1997

**Authors:** Mariëtte Goddijn, Hans G. Schipper, Lodewijk Spanjaard, Hans Wolf

**Affiliations:** 1 Department of Obstetrics and Gynecology (H4-205) Academic Medical Center University of Amsterdam PO Box 22700 Amsterdam 1100 DE The Netherlands; 2 Department of Internal Medicine Academic Medical Center Amsterdam The Netherlands; 3 Department of Medical Micobiology Academic Medical Center Amsterdam The Netherlands

## Abstract

*Background:* Little is known about fetal outcome after *Listeria monocytogenes* septicemia in the first trimester of pregnancy.

*Case:* A primigravida with *L. monocytogenes* septicemia at 9 weeks gestation was treated with amoxicillin. At 40 weeks gestation a healthy female infant was born.

*Conclusion:* This case shows that fetal outcome can be normal after appropriate treatment of maternal *L. monocytogenes* septicemia during embryogenesis.


Infectious Diseases in Obstetrics and Gynecology 5:219–221 (1997)
© 1997 Wiley-Liss, Inc.

### First Trimester *Listeria monocytogenes* Septicemia

**Mariëtte Goddijn,[1]* Hans G. Schipper,[2] Lodewijk Spanjaard,[3] and Hans Wolf[1]**

[1]*Department of Obstetrics and Gynecology, Academic Medical Center, Amsterdam, The Netherlands*
[2]*Department of Internal Medicine, Academic Medical Center, Amsterdam, The Netherlands*
[3]*Department of Medical Microbiology, Academic Medical Center, Amsterdam, The Netherlands*

Abstract

***Background:*** **Little is known about fetal outcome after *Listeria monocytogenes* septicemia in the first trimester of pregnancy.**

***Case:*** **A primigravida with *L. monocytogenes* septicemia at 9 weeks gestation was treated with amoxicillin. At 40 weeks gestation a healthy female infant was born.**

***Conclusion:*** **This case shows that fetal outcome can be normal after appropriate treatment of maternal *L. monocytogenes* septicemia during embryogenesis. Infect. Dis. Obstet. Gynecol. 5:219–221, 1997.** © 1997 Wiley-Liss, Inc.

Key words
listeria infections; listeriosis; pregnancy

Listeriosis is a rare disease. The infection is mainly found among elderly people, immunocompromised patients, neonates, or women during the perinatal period. In pregnancy, listeriosis is most often observed in the 2nd and 3rd trimesters. It may result in late abortion, stillbirth, or preterm delivery. In the neonate *Listeria monocytogenes* causes septicemia or pneumonia and occasionally meningitis.[1]

We present a case of *L. monocytogenes* septicemia at 9 weeks gestation with normal fetal outcome.

### CASE REPORT

A 29-year-old Dutch woman, gravida 1, para 0, complained about an influenza-like illness which had developed in a few hours. Being a stewardess, she visited countries in the Middle East, Africa, and Asia in the preceding 6 months. Her general practitioner considered malaria and referred her to the emergency ward.

Physical examination showed a moderately ill woman without specific signs. Her temperature was 38°C. Her uterus was soft and nontender. Laboratory examination revealed a thrombopenia (platelet count 118 × $10^9$/l) and a leukopenia with left shift (white blood cell count 4.4 × $10^9$/l with 8% bands). Malaria was excluded by a thick smear. The fever and the laboratory results were indicative of a bacterial infection. She was treated with amoxicillin 4 × 1 g (orally). One day later the blood culture yielded gram-positive rods which were identified as *L. monocytogenes* serotype 4b. The patient was admitted and amoxicillin therapy was continued intravenously (4 × 1 g). After a good recovery within 2 weeks she was discharged and amoxicillin therapy was continued for another 4 weeks (4 × 1 g orally). The most probable route of infection was the eating of feta cheese 4 days before her referral to the emergency ward.

Subsequent prenatal examinations were normal. Ultrasound screening performed at 20 and 25 weeks showed no abnormalities. At 40 0/7 weeks of gestation, a healthy female infant was born by uncomplicated vaginal delivery. The birth weight was

*Correspondence to: Dr. Mariëtte Goddijn, Department of Obstetrics and Gynecology (H4-205), Academic Medical Center, University of Amsterdam, PO Box 22700, 1100 DE Amsterdam, The Netherlands.

Received 16 October 1996

Accepted 3 March 1997

3,860 g and Apgar scores were 9 and 10 at 1 and 5 min. Surface cultures (ear, nose, and umbilical cord) were all negative as was a culture of the placenta. Histological examination of the placenta did not show typical signs of intrauterine listeria infection (granulomas, microabscesses, or vasculitis). However, at the maternal side some dystrophic calcifications were seen, marking sites of prior cell death, possibly caused by the infection.

### DISCUSSION

Little is known about *L. monocytogenes* septicemia in the first trimester of pregnancy. We found only three detailed descriptions of a first trimester *L. monocytogenes* septicemia, two with normal fetal outcome and one accompanied by spontaneous abortion. In one case a multigravida developed a *L. monocytogenes* septicemia, proved by blood culture, with possible signs and symptoms of chorioamnionitis at 13 weeks gestation. A recommended therapeutic abortion was refused by the patient. She was treated with ampicillin and gentamicin intravenously followed by trimethoprim/sulfamethoxazol orally. A healthy, culture negative female infant was delivered at term.[2] The other case was a multigravida with a *L. monocytogenes* infection at 13 weeks gestation, proved by blood culture. She had no signs of chorioamnionitis. The patient was treated with ampicillin and gentamicin intravenously. At 36 weeks gestation a cesarean section was performed because of fetal distress and a healthy boy with Apgar scores of 9 and 10 at 1 and 5 min was born.[3] The last case was treated for a *L. monocytogenes* infection, also proved by blood culture, at 12 weeks of pregnancy. There was a 2-week history of complaints. She aborted spontaneously 2 h after intravenous administration of amoxicillin was begun.[4]

In our case no clinical signs of chorioamnionitis such as abdominal tenderness were observed. An amniocentesis to verify chorioamnionitis was not performed because of the risk of this procedure at 9 weeks gestational age and absent implications for clinical management. When *L. monocytogenes* was found in the amniotic fluid we had no reason to discuss continuation of pregnancy because data about possible teratogenic effects were lacking. Placental examination was only suggestive for a prior infection, but a firm diagnosis could not be established.

Although abortion is reported as a result of maternal listeriosis,[4] convincing evidence of a causal relation is lacking. In a prospective study of 86 patients with 2 or more fetal losses endocervical cultures for *L. monocytogenes* were all negative.[5]

Listeriosis is a food-borne disease usually characterized by a flu-like illness with chills, fever, myalgia, headache, stiff neck, and back and abdominal pain.[2] Transmission is possible following consumption of unpasteurized milk, undercooked chicken, soft cheeses, and other foodstuffs. Direct transmission from animals to farmers or veterinarians is possible. Congenital infection of the fetus is induced either by hematogenous spread through the placenta or by an ascending infection from a colonized cervix.[1,6] The drug of choice is ampicillin (or amoxicillin or penicillin). In case of allergy, trimethoprim/sulfamethoxazol or erythromycin can be considered. Cephalosporins are ineffective to treat listeriosis.[1,7–9] There is no concensus on the therapeutic role of gentamicin in combination with other antimicrobial drugs.[8]

The advised dose of ampicillin or amoxicillin for adults with serious listeriosis is 6–12 g daily intravenously divided into 3–4 equal doses for at least 4 weeks. Pregnant women with mild listeriosis can be treated appropriately with ampicillin or amoxicillin 3–6 g orally for 2–3 weeks.[1] Our patient, with septicemia but mild clinical signs, was treated with amoxicillin intravenously for 2 weeks (4 × 1 g) followed by another 4 weeks of amoxicillin orally (4 × 1 g).

*L. monocytogenes* infections in the first trimester of pregnancy are rare. A maternal septicemia during embryogenesis (up to 12 weeks amenorrhea) has never been published. Our case, as well as the two other reported cases in the literature, demonstrate that fetal outcome can be normal after appropriate maternal treatment. Whether the favorable outcome depends on early treatment or an absence of placental transmission in the cases under consideration remains unsolved. Present knowledge does not provide a foundation for termination of pregnancy in case of a first trimester *L. monocytogenes* septicemia. Further case reports are needed to improve our knowledge of possible sequelae.

### REFERENCES

1. Enocksson E, Wretlind B, Sterner G, Anzen B: Listeriosis during pregnancy and in neonates. Scand J Infect Dis 71:89–94, 1990.
2. Cruikshank DP, Warenski JC: First-trimester maternal *Listeria monocytogenes* sepsis and chorioamnionitis with normal neonatal outcome. Obstet Gynecol 73:469–471, 1989.
3. Fuchs S, Hochner-Celnikier D, Shalev O: First trimester listeriosis with normal fetal outcome. Eur J Clin Microbiol Infect Dis 13:656–658, 1994.
4. Pezeshkian R, Fernando N, Carne CA, Simanowitz MD: Listeriosis in mother and fetus during the first trimester of pregnancy. Case report. Br J Obstet Gynaecol 91:85–86, 1984.
5. Mangianello PD, Yearke RR: A 10-year prospective study of women with a history of recurrent fetal losses fails to identify *Listeria monocytogenes* in the genital tract. Fertil Steril 56:781–782, 1991.
6. Shuchat A, Deaver KA, et al.: Role of foods in sporadic listeriosis. JAMA 267:2041–2045, 1992.
7. Marget W, Seeliger HPR: *Listeria monocytogenes* infections—Therapeutic possibilities and problems. Infection 16:175–177, 1988.
8. Riviera L, Dubini F, Bellotti MG: *Listeria monocytogenes* infections: The organism, its pathogenicity and antimicrobial drug susceptibility. Microbiologica 16:189–204, 1993.
9. Cherubin CE, Appleman MD, Heseltine PNR, Khayr W, Stratton CW: Epidemiological spectrum and current treatment of listeriosis. Rev Infect Dis 13:1108–1114, 1991.
